# Establishment of a novel mesenchymal stem cell-based regimen for chronic myeloid leukemia differentiation therapy

**DOI:** 10.1038/s41419-021-03499-w

**Published:** 2021-02-24

**Authors:** Shiman Zuo, Luchen Sun, Yuxin Wang, Bing Chen, Jingyue Wang, Xiangyu Ge, Yan Lu, Nanfei Yang, Pingping Shen

**Affiliations:** 1 State Key Laboratory of Pharmaceutical Biotechnology, Department of Urology, Nanjing Drum Tower Hospital, The Affiliated Hospital of Nanjing University Medical School, School of life science, Nanjing University, Nanjing, 210023 China; 2 Department of Hematology, Nanjing Drum Tower Hospital, The Affiliated Hospital of Nanjing University Medical School, Nanjing, 210008 China; 3 Department of Pathology, Yale University School of Medicine, New Haven, CT 06510 USA

**Keywords:** Chronic myeloid leukaemia, Mesenchymal stem cells

## Abstract

Chronic myeloid leukemia (CML) is characterized by the accumulation of malignant and immature white blood cells which spread to the peripheral blood and other tissues/organs. Despite the fact that current tyrosine kinase inhibitors (TKIs) are capable of achieving the complete remission by reducing the tumor burden, severe adverse effects often occur in CML patients treated with TKIs. The differentiation therapy exhibits therapeutic potential to improve cure rates in leukemia, as evidenced by the striking success of all-trans-retinoic acid in acute promyelocytic leukemia treatment. However, there is still a lack of efficient differentiation therapy strategy in CML. Here we showed that *MPL*, which encodes the thrombopoietin receptor driving the development of hematopoietic stem/progenitor cells, decreased along with the progression of CML. We first elucidated that MPL signaling blockade impeded the megakaryocytic differentiation and contributed to the progression of CML. While allogeneic human umbilical cord-derived mesenchymal stem cells (UC-MSCs) treatment efficiently promoted megakaryocytic lineage differentiation of CML cells through restoring the *MPL* expression and activating MPL signaling. UC-MSCs in combination with eltrombopag, a non-peptide MPL agonist, further activated JAK/STAT and MAPK signaling pathways through MPL and exerted a synergetic effect on enhancing CML cell differentiation. The established combinational treatment not only markedly reduced the CML burden but also significantly eliminated CML cells in a xenograft CML model. We provided a new molecular insight of thrombopoietin (TPO) and MPL signaling in MSCs-mediated megakaryocytic differentiation of CML cells. Furthermore, a novel anti-CML treatment regimen that uses the combination of UC-MSCs and eltrombopag shows therapeutic potential to overcome the differentiation blockade in CML.

## Introduction

Chronic myeloid leukemia (CML) is a typical stem cell disorder characterized by the formation of the fusion protein—tyrosine kinase BCR-ABL in hematopoietic stem cells (HSCs), which leads to the malignant transition of HSCs to leukemic stem cells (LSCs)^1^. As a pathological result of tumorigenesis, differentiation of CML cells is arrested and immature myeloid blasts accumulate in the bone marrow and peripheral blood, impairing various biological processes such as hematopoiesis, metabolism, and immune response^2^. Currently, selectively targeting BCR-ABL kinase is the standard of care for CML using tyrosine kinase inhibitors (TKIs) including imatinib, dasatinib, and nilotinib, and has manifested clinical benefits in improving patients’ survival^3,4^. However, due to the persistence of leukemic stem cells (LSCs) independent of BCR-ABL, CML relapses occur in ∼61% of patients after TKI withdrawal, and few patients achieve complete molecular response (CMR)^5,6^. These despondent outcomes highlight the urgent need for more efficacious therapies in treating CML.

The tactic that induces malignant cells to overcome the barrier of differentiation, also termed as pro-differentiation therapy, provides a rational avenue for combating with the blood carcinoma. It has been successfully applied to treat acute myeloid leukemia (AML)^7^. Considering the differentiation hierarchy of CML cells stagnates in the stage between stem cells and progenitor cells, the leukemic cells have been shown to be compelled to the megakaryocytic lineage determination, the precursor of blood platelet, which further ameliorates malignancy of LSCs in CML^8,9^. In fact, TKI-targeted therapy induces megakaryocytic differentiation in human CML cells, which might be partially attributed to the induction of MPL expression, a crucial cytokine receptor in megakaryocytic fate determination^10–12^. Nevertheless, compared to normal HSCs, CML-derived LSCs are less sensitive to TPO stimulation^13^. A possible reason is the damaged TPO/MPL signaling transduction cascade in CML cells during the malignant transformation. Thus, restoring MPL signaling in CML cells is essential for establishing the pro-differentiation therapeutic strategies.

Adoptive transfer of mesenchymal stem cells (MSCs) has been proposed as an effective way to treat certain vicious diseases, including malignant blood disorders^14,15^. In clinical trials, MSCs transplantation (MSCT) inhibits CML cell proliferation and attenuates the CML progression^16^. Besides, MSC is the key mediator in regulating megakaryocytic differentiation of HSCs—MSCs are able to secret TPO, as well as some other functional factors to remodel epigenetic program for determining the megakaryocytes (MKs) fate in HSCs^17–19^. This evidence supports the idea that MSCs treatment is an effective approach to restore the megakaryocytic differentiation response in CML cells.

In this study, we demonstrated that human CML cells gradually lose the expression of *MPL* during CML progression, which explains the less efficiency of megakaryocytic differentiation in CML cells. We also showed that umbilical cord-derived MSCs (UC-MSCs) treatment rescued *MPL* expression and further promoted the megakaryocytic differentiation in CML cells. The pro-differentiation effect of UC-MSCs might result from their ability to activate JAK/STAT and p38 MAPK signaling. Moreover, the MPL agonist eltrombopag exhibited a synergistic effect combining with UC-MSCs on inducing CML cell differentiation and protected CML mice from severe adverse effects. Therefore, we proposed a novel approach for CML treatment by combining UC-MSCs with TPO signaling restoration.

## Methods

### Cell culture

Human umbilical cord-derived mesenchymal stem cells (UC-MSCs) were separated from human umbilical cord obtained from Nanjing Drum Tower Hospital (Nanjing, China) after informed consent was obtained. UC-MSCs were cultured in MEM-α Medium (BasalMedia, China). UC-MSCs used in all experiments were controlled within passage 10. K562 cells and LAMA-84 cells were obtained from China Center for Type Culture Collection (Wuhan, China) and cultured in RPMI 1640 medium (BasalMedia, China). All media were supplemented with 10% fetal bovine serum, penicillin (100 U/mL), streptomycin (100 μg/mL), and maintained in a humidified incubator at 37 °C under 5% CO_2_.

### Microarray data processing

Several previously published datasets were used for gene expression profiles, including GSE13159, GSE24739, GSE47927. Raw data were directly downloaded from Gene Expression Omnibus (GEO) database. Raw data were normalized by robust multi-array average (RMA) method, R package limma^20^ (v3.44.3) was used for identifying differentially expressed genes (DEGs). Absolute log2 fold change (log_2_FC) >1 and *P* value <0.05 was considered statistically significant, unless otherwise indicated. All data were processed using the R software (v4.0.2), while statistical analysis of *MPL* expression levels was performed by GraphPad Prism (v8.0).

### Gene set enrichment analysis

Pathways (KEGG database) gene set enrichment analyses were performed by clusterProfiler (v3.16.1) R package^21^. The gene-sets were derived from the Broad institute’s Molecular Signatures Database (MSigDB v7.1).

### Weighted gene co-expression network analysis

Weighted gene co-expression network analysis (WGCNA) was performed by WGCNA (v1.69) R package^22^ to identify the gene modules. Briefly, raw data of GSE47927 were downloaded from GEO database and normalized by RMA method. A suitable soft threshold of six was selected. The network was constructed and modules whose correlation greater than 0.7 were merged together. Finally, 25 modules were identified. R package clusterProfiler^21^ (v3.16.1) was used for Gene ontology (GO) enrichment analysis and upstream transcriptional regulators identification of interesting gene modules.

### Quantitative real-time PCR

Total RNA was isolated from cells and reverse transcription was performed with 5× All-In-One RT MasterMix (Abmgood, Canada) kit. Quantitative RT-PCR was performed on a C1000 PCR System (Bio-Rad) using SYBR Green Master Mix (Vazyme, China) kit. The expression of the measured genes in each sample was normalized to *GAPDH* mRNA expression levels. Primers used for qPCR are shown in Table S1.

### Cell cycle, proliferation, and flow cytometric analysis

To evaluate cell cycling, co-cultured K562 cells were fixed with 70% ethanol in PBS at −20 °C overnight. Propidium iodide (Sigma-Aldrich, USA) was used as a nucleic acid dye. To measure cell proliferation rate, CFSE (Sigma-Aldrich, USA) staining was performed according to the manufacturer’s protocols. Levels of CD41 (Biolegend), CD42b (Biolegend), and MPL (Bioss) were also analyzed by flow cytometry. Flow cytometric analysis was performed on a NovoCyte Flow Cytometer (ACEA Biosciences Inc). The statistics presented are based on at least 10,000 events gated on the population of interest.

### Wright–giemsa staining

Cells were prepared onto a microscope slide and allowed to air dry. Slides were stained with Wright-Giemsa reagents (Jiancheng, China). Stained cells were rinsed in deionized water, and coverslips were affixed prior to microscopy.

### Small interfering RNA (siRNA) transfection

MPL siRNA (GenePharma, China) and negative control siRNA (NC-siRNA) were used to examine the megakaryocytic differentiation effect. About 1 × 10^5^ K562 cells were transfected with 30 pmol of MPL siRNA or NC siRNA using Lipofectamine 2000 (Invitrogen, USA) and Opti-MEM (Gibco, USA) according to the manufacturer’s recommendations. After 24 h, the cells were co-cultured with MSCs for an additional 48 h. Then, an aliquot of live cells was collected for RT-PCR and the remaining cells were analyzed by western blot. The assay methods were described above.

### Adenovirus infection

The adenovirus Ad-mCherry-GFP-LC3B was purchased from Beyotime Biotechnology (China). K562 cells were infected with adenoviral vectors at a multiplicity of infection (MOI) of 20 and treated with experimental conditions as indicated.

### Lentiviral plasmids production and cell infection

The cDNA encoding MPL was cloned, and inserted into the pLenti6/v5 lentiviral vector. The lentiviral particles were produced in HEK293T cells by co-transfecting the MPL plasmid with two package plasmids (psPAX2 and pMD2.G) using Lipofectamine 2000 (Invitrogen, USA). Medium containing concentrated lentivirus (by using Lenti-Concentin Virus Precipitation Solution, Cat. no. C103-01) was used to infect K562 or LAMA-84 cells. After 3 days transduction, cells were enriched by blasticidin for another 3 days and then cells were harvested.

### Western blot analysis

After treatment, cells were harvested and proteins were extracted by whole-cell lysis (Beyotime, China). Cell membrane protein was isolated by Membrane and Cytosol Protein Extraction Kit (Beyotime, China). Protein concentration was determined by BCA reagent. Lysates were resolved by SDS-polyacrylamide gels and transferred to PVDF membranes. Antibodies against the following proteins were used as probes: p38 MAPK (#9212), p-p38 MAPK (T180/Y182) (#9215), and p-STAT3 (Y705) (#9145) were purchased from Cell Signaling Technology. STAT3 (BS1336), ERK (BS1112), JAK2 (BS2432), p-ERK (T202/Y204) (BS5016), and p-JAK2 (Y1007/Y1008) (BS94058) were obtained from Bioworld. LC3A/B (GB11124) was purchased from Servicebio. MPL (220651), AKT (342529), and β-actin (382299) were supported by Zen-Bioscience. p-AKT (S473) (AP3434a) was obtained from Abcepta. GAPDH (AC035) was purchased from ABclonal. ATPase Na^+^/K^+^ beta 2 (bs-1152R) was purchased from Bioss. Signals were detected by a chemiluminescence System (Clinx) using High-sig ECL Western Blotting Substrate (Tanon, China). The intensities of the bands were quantified by Image J software.

### Xenograft experiments

The xenograft leukemia model was established by intravenous injection of K562 cells (5 × 10^7^/mouse) into the tail veins of nude mice of 6–8 weeks old. One week after transplantation, the mice were grouped and treated with PBS (*n* = 6), eltrombopag (*n* = 6), MSCs (*n* = 8), and MSCs combined with eltrombopag (*n* = 8) respectively. MSCs (1 × 10^6^/mouse) were injected intravenously once a week. Eltrombopag (25 mg/kg) was injected intraperitoneally every other day for 6 weeks. Animal welfare and experimental procedures were carried out in strict accordance with the Guide for the Care and Use of Laboratory Animals (The Ministry of Science and Technology of China) and the related ethical regulations of our university.

### HE staining and immunofluorescence

Mice were sacrificed and spleens were isolated. Formalin-fixed, paraffin-embedded samples were segmented and stained with hematoxylin and eosin (HE). The pathologic changes of spleen were observed by the optical microscope (Nikon). The tissue sections (4 μm) were deparaffinized and rehydrated through xylene and graded alcohols. Tissue sections were boiled in citrate buffer for antigen retrieval, and blocked with 1% serum goat for 60 min. The slides were stained with the primary antibody anti-CD45 (Santa Cruz) at 4 °C overnight and the secondary Alexa Fluor 488-labeled goat anti-mouse IgG antibodies (Beyotime, China) at room temperature for 2 h. Nuclei were counterstained with DAPI. Images were acquired using a Nikon A1R confocal laser scanning microscope.

### Statistics

Statistical analysis was performed using GraphPad Prism software (v8.0). Error bars indicated the Standard Error of Mean (SEM). Differences were analyzed by unpaired Student’s t test or one-way ANOVA depending on experimental conditions. *P* values ≤0.05 were considered statistically significant. All statistical analysis was generated based on at least three independent experiments.

## Results

### MPL signaling was abrogated in CML cells

**Fig. 1:**
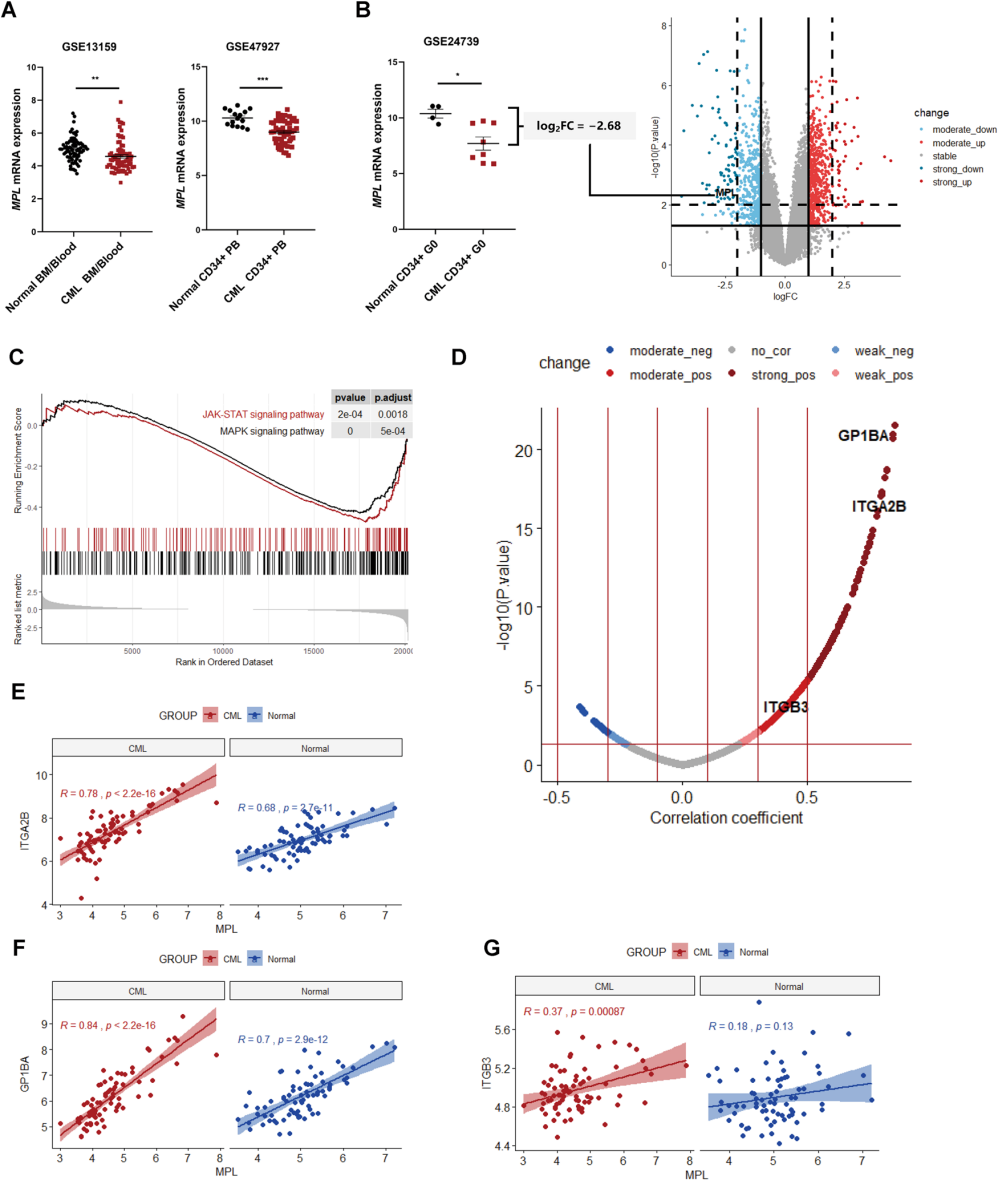
MPL was involved in differentiation arrest of CML cells. **A** *MPL* expression levels of CML samples and their normal controls in GEO accession including GSE13159 (*n*_normal_ = 74, *n*_CML_ = 76), GSE47927(*n*_normal_ = 15, *n*_CML_ = 52). **B** *MPL* expression levels and volcano plot of differential gene profiles between normal (*n* = 4) and CML (*n* = 8) CD34^+^ quiescent cells (GSE24739). Criteria to filter DEGs were absolute log_2_FC >1 & *P* value <0.05 (as the solid lines showed), the high-threshold to shrink DEGs was defined as absolute log_2_FC >2 & *P* value <0.01 (as the dashed lines showed). MPL was labeled in the volcano plot. **C** GSEA showing negative enrichment of JAK/STAT, MAPK pathways gene signatures. Genes were ranked by log_2_FC. **D** Volcano plot of correlations between *MPL* and the other detected genes in CML samples (GSE13159, *n* = 76). X-axis represented the Pearson coefficients and the Y-axis showed negative log10 of the *P* values. The correlations between MPL and three MK marker genes, including *ITGA2B*, *GP1BA,* and *ITGB3*, were labeled in the volcano plot and further visualized by correlation plots (**E**–**G**). Error bars indicated mean ± standard error of the mean (SEM) (*P* values: two-tailed Student’ s *t* test; *P <0.05, **P <0.01, ***P <0.001).

Although it has been observed TKI treatment increased the level of TPO in Chronic myeloid leukemia (CML) patients^23^, the relation between TPO/MPL pathway activation and CML progression remains unclear. Here we found that, compared to normal samples, there was a significantly decreased expression of TPO receptor (*MPL*) in CML samples (Fig. 1A, B), particularly in CD34^+^ quiescent cells (Fig. 1B). We further compared the differential expressed genes (DEGs) rigidly and clustered the MPL signaling-related pathways between normal and CML cells (GSE24739). Even though a high threshold was set up for filtering the DEGs, *MPL* was (log_2_FC = −2.68 and *P* value = 0.006) still gated in the area containing the marked downregulated genes (Fig. 1B). Gene set enrichment analysis (GSEA) revealed that MPL signaling, including JAK/STAT and MAPK pathways, was dramatically impeded (Fig. 1C). These data demonstrated that MPL signaling might play a critical role in the phenotypic dysfunctions of CML cells.

Next, we aimed to establish a direct link between MPL-mediated differentiation arrest and CML progression. We first dissected the *MPL* mRNA abundance in HSCs and diverse hematopoietic progenitor cells (HPCs). Among these cells, megakaryocyte-erythroid progenitors (MEPs) and multipotent progenitor cells (MPPs) showed a marked decrease of *MPL* gene expression (Fig. S1A). Moreover, the *MPL* expression in every type of progenitor was inversely correlated to the progressive phases of disease (Fig. S1B), indicating that the regulatory hindrance in activating MPL signaling in progenitors drove the malignant transformation in CML progression. To further confirm the tight correlation between CML progression and MPL gene expression, we performed weighted gene co-expression network analysis (WGCNA) across samples from three normal donors, six CP, four AP, and two BC patients. We first conducted the preliminary sample clustering and calculated the Euclidean distance among all samples (Fig. S2A). Next, we used the soft-thresholding method to define the gene co-expression correlation in the network. Figure S2B showed that when the soft-threshold power was chosen as six, two correlation parameters—scale-free fit index was greater than 0.85, and the mean connectivity declined to the baseline. These parameter control management successfully helped us to clustered 32 epigengenes (Fig. S2C), which represented the co-expression patterns in each module group. By using the dynamic branch cutting approach, we further merged the gene modules that have similar gene expression profiles (the cutting baseline was 0.7 height) (Fig. S2D). Finally, WGCNA grouped genes with similar co-expression patterns into 25 modules (Fig. S2E). We found that *MPL* gene was grouped into the yellow module. In normal samples, the yellow module membership value was 0.4, suggesting the *MPL* gene expression was positively correlated with the normal hematopoietic function. However, this value was changed to −0.23 in BC phase samples, verifying the negative relationship between *MPL* gene expression and CML progression (Fig. S2E). Furthermore, the GO analysis confirmed that the HSPCs differentiation process-related genes were highly enriched in the yellow module (Fig. S2F). Moreover, we also enriched the upstream transcriptional factors that regulated the genes in the yellow module. The results showed that the TAL1 and STAT1, two key transcription factors involved in the megakaryocyte-erythroid differentiation process, were obtained (Fig. S2G). Furthermore, correlation analysis on the CML samples from microarray innovations in LEukemia (MILE) project^24,25^ indicated that 153 genes showed a strong positive correlation and 486 genes moderate positively correlated to *MPL* expression (Fig. 1D). Among them, *ITGA2B*, *GP1BA*, and *ITGB3* (also known as *CD41*, *CD42b*, and *CD61,* respectively), the megakaryocyte markers, were co-expressed with *MPL* (Fig. 1E–G).

All these data demonstrated that MPL signaling repression impeded the megakaryocytic differentiation in CML cells and contributed to the cancer progression.

### UC-MSCs-induced megakaryocytic differentiation in CML cells

**Fig. 2:**
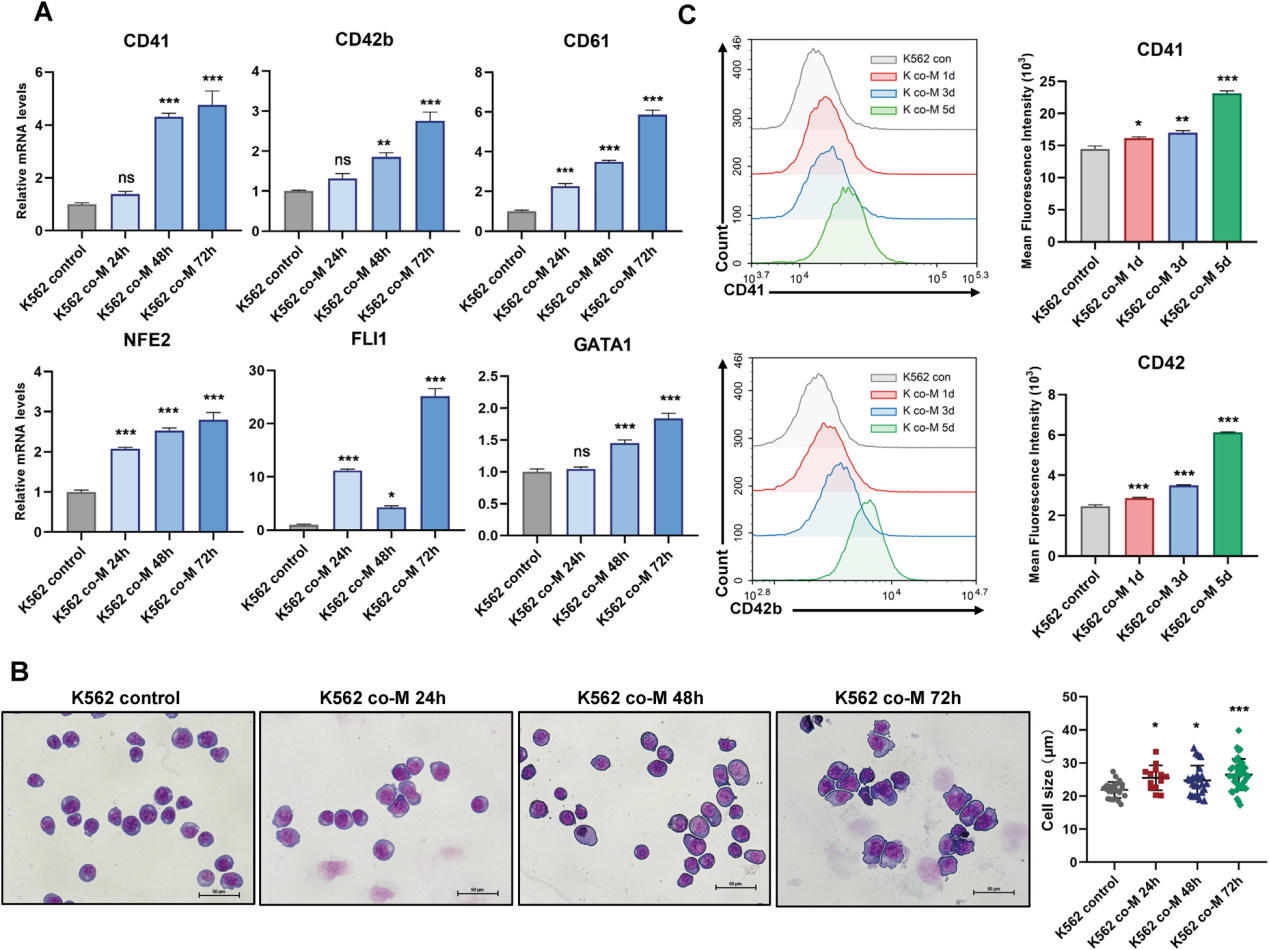
MSCs-induced profound differentiation in CML cell line. **A** K562 cells were cultured alone or co-cultured with MSCs for different times. The relative mRNA levels of differentiation genes and transcription factors were assessed by RT-qPCR (*n* = 3). **B** Morphological changes accompanied by megakaryocytic differentiation were confirmed by Wright–Giemsa staining of cells with or without MSCs culture. Scale bars, 50 μm. Comparison of the cell size in K562 cells prior to or after stimulation by MSCs. **C** The amounts of cell surface protein CD41 and CD42b were measured by flow cytometric. The amounts of K562 cell surface proteins were expressed as the geometric mean fluorescence intensity. All statistical data in this figure represented the mean ± SEM (*P <0.05, **P <0.01, ***P <0.001, ns, not significant, one-way ANOVA for multiple comparisons). M MSCs.

Umbilical cord-derived MSCs (UC-MSCs, hereafter called MSCs) were able to promote the differentiation of malignant proliferating cells^26,27^. We were curious about whether MSCs had the potential to drive CML cell differentiation. To evaluate our hypothesis, we first conducted the experiment that employed MSCs to treat CML cells and detected the expression of megakaryocyte-associated makers. The results suggested that mRNA levels of typical megakaryocytic differentiation makers and transcription factors, including *CD41*, *CD42b*, *CD61*, *NFE2*, *GATA1*, and *FLI1*, were significantly upregulated in CML cell lines (K562, LAMA-84 cells) (Figs. 2A and S3A). Meanwhile, K562 cells treated by MSCs resulted in the loss of expression of erythroid genes including *HBA1*, *HBB*, *HBE1*, and *KLF1* (Fig. S3D), suggesting that MSCs boosted megakaryocytic-biased differentiation of CML cells. Biased differentiation was further confirmed by Wright–Giemsa staining and FACS analysis. Wright–Giemsa staining showed enlarged cell size, which was reminiscent of megakaryocyte formation (Figs. 2B and S3B). FACS analysis showed that CD41 and CD42b, the surface markers of megakaryocyte, increased on CML cells during co-culturing (Figs. 2C, S3C and S3E). Parallelly, FACS analysis indicated that MSCs led to the increased accumulation in G0/G1 cell cycle (Fig. S4A) and reduced proliferation rates (Figs. S4B and S4C) in K562 cells. These findings suggested that MSCs-mediated cell differentiation and conferred these aberrant blood cells with megakaryocyte-like phenotypes.

Since autophagy has been proposed to play a role in megakaryocytic differentiation of K562 cells^28^, we investigated whether megakaryocytic differentiation induced by MSCs in K562 cells was associated with autophagy induction. RT-qPCR showed that MSCs-mediated differentiation accompanied by increasing expressions of autophagy-related genes (ATGs) and microtubule-associated protein light chain 3 (LC3) in K562 cells (Fig. S5A). MSCs mediated autophagy involved the conversion of LC3 from the LC3B-I to LC3B-II form (Fig. S5B). Meanwhile, the adenovirus was used to track autophagosome marker LC3 in K562 cells. We observed a slight increase in LC3-II levels upon MSCs treatment (Fig. S5C). These results manifested that MSCs-treated cells had undergone an autophagic process. Moreover, to determine the direct relationship between MSCs-mediated differentiation and autophagy induction, autophagy was directly induced by glucose deprivation or serum starvation, we found that megakaryocytic differentiation genes expressions were markedly upregulated (Fig. S5D). On the other hand, MSCs-induced megakaryocytic differentiation of K562 cells was attenuated by using the autophagy inhibitor 3-methyladenine (3-MA) to some extent. As shown in Fig. S5E, F, the differentiation makers decreased when treated with 3-MA. Morphological observation showed extensive cytoplasmic vacuole accumulation in MSCs-treated cells. However, the MSCs-mediated morphological changes were abolished after treatment with 3-MA (Fig. S5G). These data corroborated that autophagy was involved in MSCs-induced megakaryocytic differentiation of K562 cells.

### MSCs restored impaired MPL signaling in CML cells

**Fig. 3:**
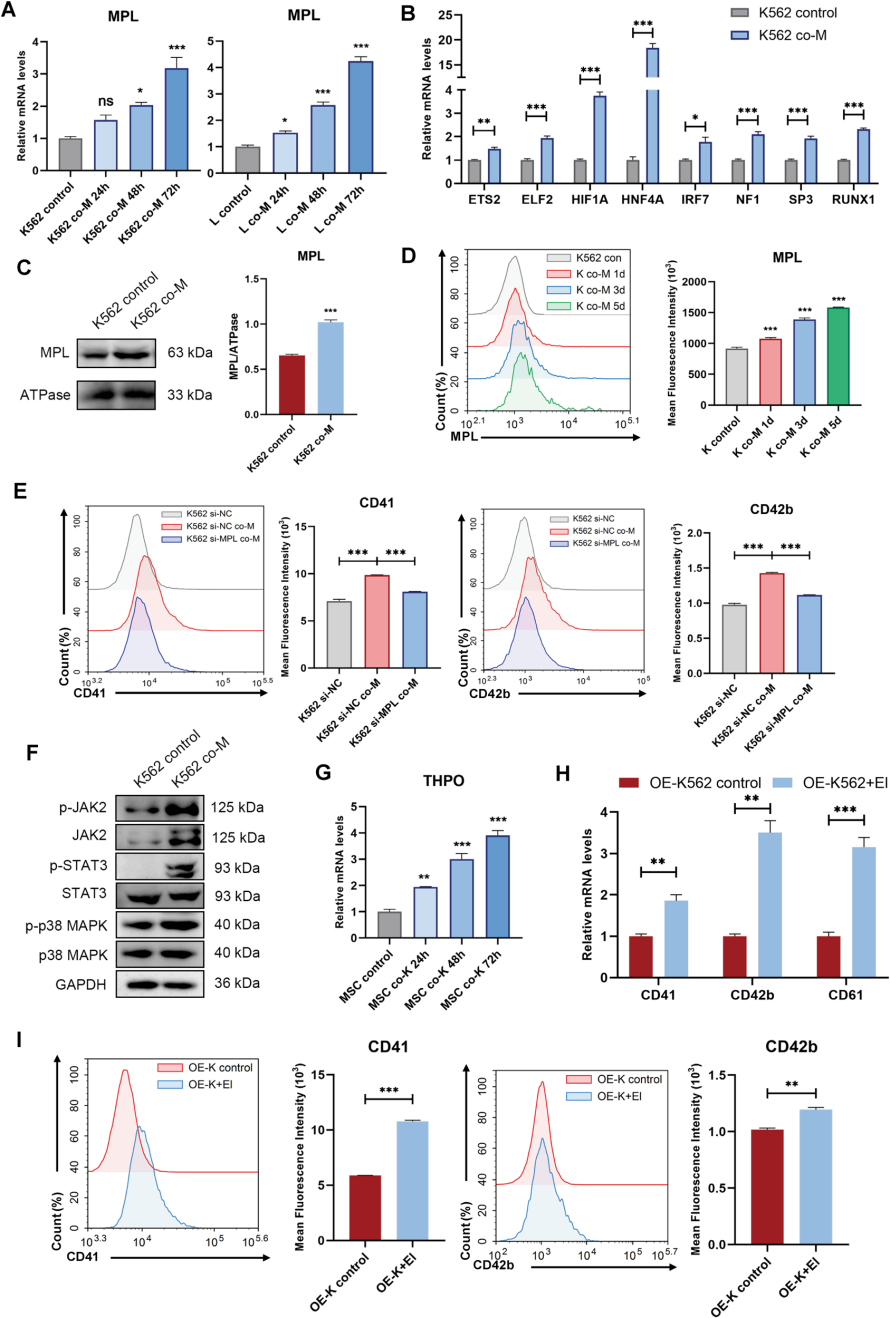
MSCs-induced megakaryocytic differentiation through restoring *MPL* expressions of CML cells. **A** K562 cells (left) and LAMA-84 cells (right) were co-cultured with MSCs, respectively. The expression levels of *MPL* were measured by RT-qPCR (*n* = 3). **B** K562 cells were co-cultured with MSCs for 48 h, the expression of upstream transcription factors of MPL in K562 cells was assessed by RT-qPCR (*n* = 3). **C** K562 cells were treated with MSCs for 48 h, the cell-surface expression of MPL was measured by western blotting. ATPase Na^+^/K^+^ beta 2 (ATPase) was used as a loading control for membrane fraction. **D** K562 cells were cultured alone or co-cultured with MSCs for the indicated time. MPL levels were measured by flow cytometric. **E** K562 cells were transfected with siRNA negative control (K562 si-NC) or MPL-siRNA (K562 si-MPL). The K562 si-NC or K562 si-MPL cells were co-cultured with MSCs. After 48 h, the cell surface protein CD41 and CD42b in K562 cells were analyzed by flow cytometric. **F** The phosphorylation status of correlated signaling pathways was determined by western blotting after treatment with MSCs for 48 h. GAPDH was used as a loading control. **G** The expression levels of *THPO* in MSCs were assessed by RT-qPCR (*n* = 3). **H** K562 cells were overexpressed MPL (OE-K562), the relative mRNA levels of differentiation genes were assessed by RT-qPCR after treatment with El for 48 h. **I** CD41 and CD42b levels of OE-K562 cells were measured by flow cytometric after treatment with El for 48 h. All statistical data in this figure represent the mean ± SEM (*P <0.05, **P <0.01, ***P <0.001, ns, not significant, by one-way ANOVA in **A**, **D**, and **G** by Student’s *t* test in **B**, **C**, **E**, **H**, and **I**).

Given the tight link of MPL pathway and CML progression, we speculated that the activation of MPL signaling was involved in the MSCs that guided the megakaryocytic differentiation in CML cells. The RT-qPCR assay showed that the expressions of *MPL*, as well as its upstream transcription factors, were notably increased in K562 cells and LAMA-84 cells after co-cultured with MSCs (Fig. 3A, B). Similarly, western blot analysis and FACS analysis indicated that MSCs promoted cell-surface expression of MPL (Figs. 3C, D and S6A). Next, MPL knockdown K562 cells (K652 si-MPL) and negative control K562 cells (K562 si-NC) were generated by specific siRNA transfection (Fig. S6B), and the genetic editing cells were treated with MSCs. As shown in Fig. 3E and Fig. S6C, the expression levels of CD41 and CD42b in K562 si-MPL were significantly reduced when compared with that of K562 si-NC cells, suggesting that MPL directly controlled the MSC-mediated differentiation. We found that JAK2, STAT3, and p38, key nodes in MPL signaling pathways, showed an increased level of phosphorylation. (Figs. 3F and S6D). Besides, *THPO* gene transcription was robustly activated (fourfold approximately) in co-incubated MSCs (Fig. 3G). To confirm that the MPL pathway is involved in the differentiation process, we ectopically overexpressed MPL in K562 cells (OE-K562 cells) and LAMA-84 cells (OE-LAMA cells) (Fig. S6E, F). The expressions of differentiation markers in OE-K562/OE-LAMA cells were determined by RT-qPCR and flow cytometry in the presence or absence of MPL aganisit, eltrombopag. Figures 3H, I and S6G showed eltrombopag significantly promoted OE-K562 and OE-LAMA cells megakaryocytes differentiation. Together, these results strongly manifested that MSCs induced the CML cell differentiation via the restoration of damaged MPL signaling in cancer cells.

### MSCs initiated the response of CML cell to MPL agonist

**Fig. 4:**
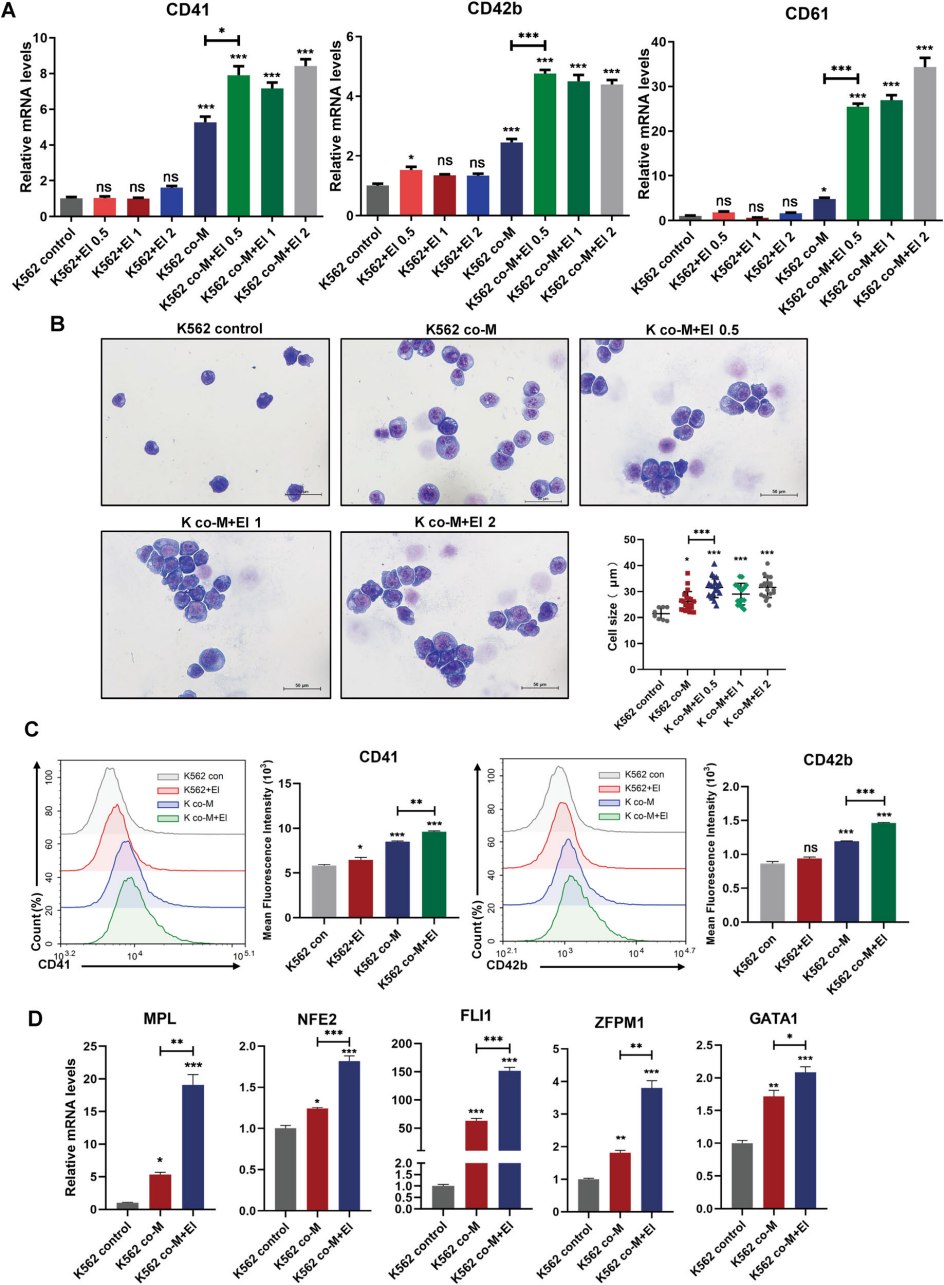
Combination treatment with MSCs and El triggered synergistically differentiation effects in K562 cells. **A** K562 cells were cultured alone or co-cultured with MSCs in the presence or absence of different doses of El (0.5 μM, 1 μM, and 2 μM) for 48 h. The relative gene expression of differentiation genes was assessed by RT-qPCR (*n* = 3). **B** Morphological changes in K562 cells that accompanied megakaryocytic differentiation were confirmed by Wright–Giemsa staining of cells in the presence and absence of El (0.5 μM, 1 μM, and 2 μM) for 48 h. Scale bars, 50 μm. **C** The expression of surface markers CD41 and CD42b of K562 cells was measured by flow cytometric analysis and the mean fluorescence intensity was quantified. **D** K562 cells were treated with either MSCs or combination with El for 48 h, the expression levels of megakaryocytic differentiation transcription factors were assessed by RT-qPCR (*n* = 3). Data are represented as the mean ± SEM (*P <0.05, **P <0.01, or ***P <0.001, ns, not significant, one-way ANOVA was used for multiple comparisons). El eltrombopag.

We next asked if MSCs could enhance the efficiency of MPL agonist—eltrombopag (El) on CML cells^29^. Unexpectedly, although the treatment of El alone failed to induce the differentiation of CML cells, the combination of MSCs and El did trigger synergistic megakaryocytic differentiation in K562 cells, substantiated by higher expression levels of *CD41*, *CD42*, and *CD61*, as well as increased cell size than MSCs treatment alone (Fig. 4A, B). Consistently, flow cytometry analysis revealed the increased expression of CD41 and CD42b in K562 cells with the combinatory regimen compared to MSCs or El alone (Fig. 4C). Similar results were also obtained in LAMA-84 cells (Fig. S7A, B). The pro-megakaryopoiesis effect of MSCs in combination with El was further verified by increased levels of the expression of *MPL* and transcription factors such as *NFE2*, *FLI1*, *ZFPM1*, and *GATA1* in K562 and LAMA-84 cells after the combinational treatment (Figs. 4D and S7C). Taken together, these findings demonstrated that MSCs synergistically facilitated CML cell differentiation with El-mediated MPL agonism.

### Combined treatment with MSCs and El synergistically promoted CML cell differentiation in vivo

**Fig. 5:**
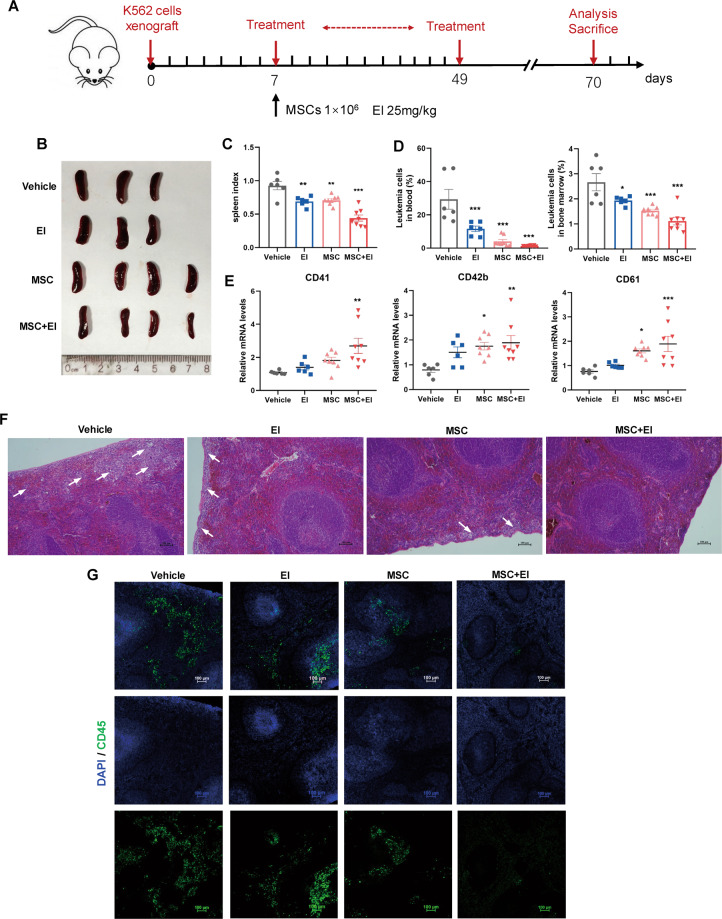
El potentiated the efficacy of MSCs on anti-leukemia in vivo. **A** Schematic illustration of experimental design. **B** Representative images of spleens from four groups. **C** The weight of spleens in different groups. **D** Percentage of human CD45^+^ leukemia cells in peripheral blood (PB) and bone marrow (BM) in vehicle and treatment mice after 10 weeks of transplantation. **E** Human CD45^+^ cells from a single-cell suspension of peripheral blood were sorted using FASC. RT-qPCR analysis on FACS-isolated human CD45^+^ cells for CD41, CD42b, CD61. **F** Histologic sections of spleen were stained with hematoxylin-eosin. Scale bars, 100 μm. **G** Representative infiltration of CML cells in the spleen as observed via immunofluorescence with anti-CD45 antibody (green). Nuclei are stained with DAPI (blue). Scale bars, 100 μm. All statistical data in this figure showed the mean ± SEM (*P <0.05, **P <0.01, ***P <0.001; one-way ANOVA was used for multiple comparisons). El eltrombopag.

To assess in vivo efficacy of the MSCs and El combination in a xenograft leukemia model. Nude mice bearing xenografts of K562 cells were treated with either MSCs or El alone or the combination regimen (Fig. 5A). As shown in Fig. 5B, C, mice in vehicle group had more severe splenomegaly compared with combination group. It was noteworthy that the leukemia burden of the combination-treated mice decreased to 2% in PB and 1% in BM (Fig. 5D), which was associated with a markedly higher level of differentiation than that associated with the single-agent treatments, as supported by the upregulation of the differentiation markers *CD41*, *CD42b*, and *CD61* (Fig. 5E). Furthermore, histological analysis showed a significant attenuation of splenic infiltration of leukemia cells in combination-treated mice (Fig. 5F, G). Collectively, these results proved that MSC/El treatment efficiently ameliorated the CML burden and executed a synergistic differentiation-promoting effect in vivo.

### MSCs and El-induced K562 cells megakaryocytic differentiation via JAK/STAT and MAPK signal pathway

**Fig. 6:**
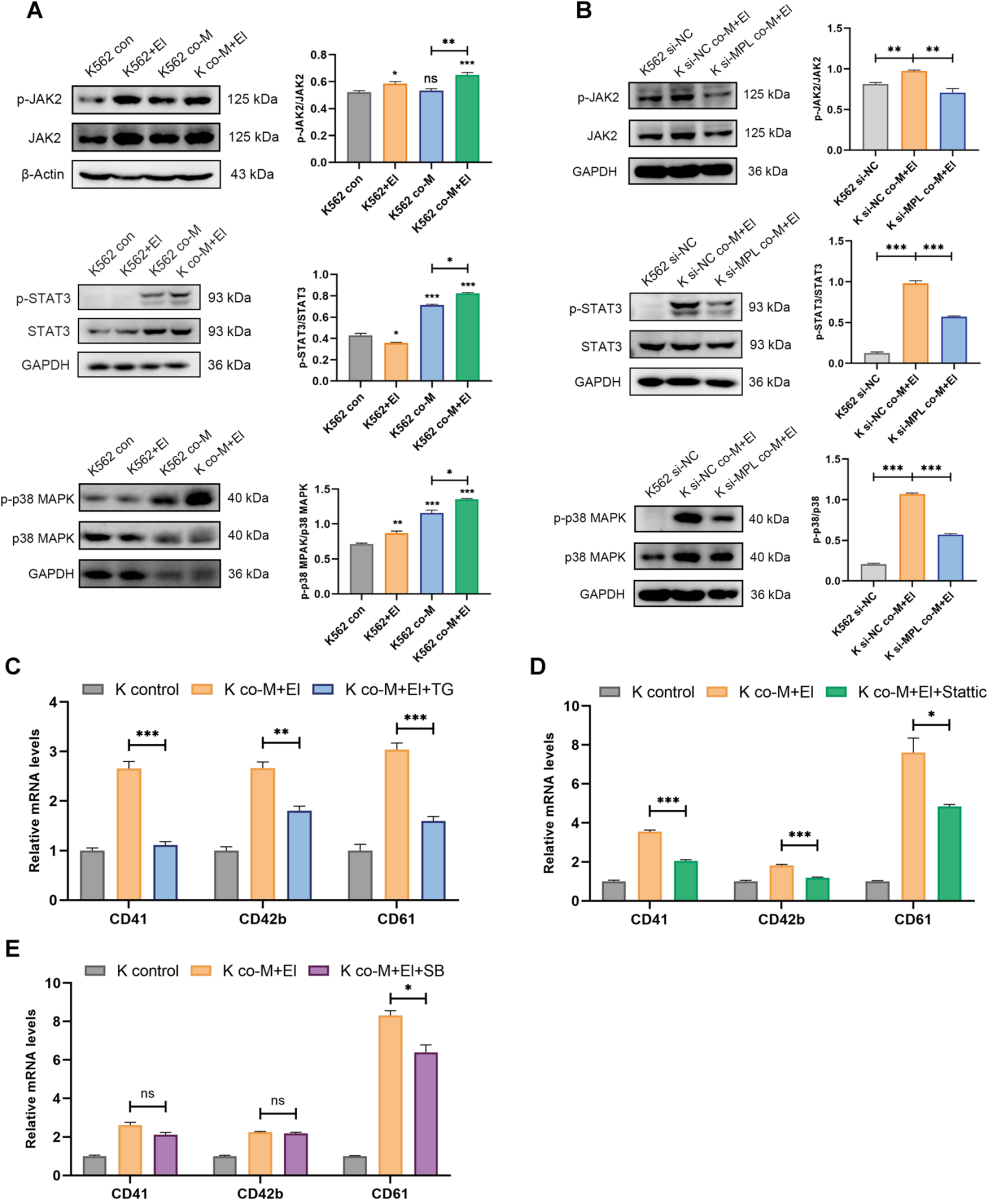
MSCs and El-activated CML cell differentiation through JAK2/STAT3 and p38 MAPK pathways. **A** The protein levels of correlated signaling pathways and phosphorylation status were measured by western blotting after treatment with either MSCs or El alone or the combination for 48 h. GAPDH or β-Actin was used as a control. **B** K562 cells were transfected with siRNA negative control (K562 si-NC) or MPL-siRNA (K562 si-MPL). The K562 si-NC or K562 si-MPL cells were cultured alone or co-cultured with MSCs and El. After 2 days, protein levels of correlated signaling pathways and phosphorylation status were measured by Western blotting. GAPDH was used as a control. **C** K562 cells were treated with the JAK2 inhibitor TG101209 (TG) (1 μM) followed by co-culturing with MSCs for 48 h. The mRNA expression of differentiation markers was determined by RT-PCR (*n* = 3). **D** K562 cells were treated with the STAT3 inhibitor Stattic (5 μM) followed by co-culturing with MSCs for 48 h. The mRNA expression of differentiation markers was determined by RT-PCR (*n* = 3). **E** K562 cells were treated with the p38 MAPK inhibitor SB239063 (SB) (10 μM) followed by co-culturing with MSCs for 48 h. The mRNA expression of differentiation markers was determined by RT-PCR (*n* = 3). All statistical data in this figure represented the mean ± SEM (*P <0.05, **P <0.01, ***P <0.001, ns, not significant, by one-way ANOVA in **A** and by Student’s *t* test in **B**–**E**). El: eltrombopag, TG: TG101209, SB: SB239063.

JAK/STAT, MAPK pathways were well-characterized MPL downstream signaling, which was validated to be downregulated in the CML cells (Fig. 1C). To elucidate the underlying intracellular pathways involved in combinational treatment-mediated megakaryocytic differentiation, K562 cells were co-cultured with MSCs in the presence of El. The activation of the downstream signaling of TPO/MPL axis was detected. The results showed that MSCs and El combination treatment robustly induced phosphorylation of JAK2, STAT3, and p38 MAPK (Fig. 6A). Meanwhile, phosphorylation levels of AKT and ERK were not altered (Fig. S8A). These observations indicated that MSCs in combination with El synergistically activated certain MPL downstream signaling. Furthermore, in the MPL knockdown cells, the activation of JAK2/STAT3 and p38 MAPK signaling was markedly attenuated (Fig. 6B). Moreover, cells were treated with specific inhibitors targeting JAK2 (TG101209), STAT3 (Stattic), and p38 MAPK (SB239063), and MK markers were detected. The results indicated that combinational treatment-mediated megakaryocytic differentiation would be markedly abrogated by these inhibitors (Fig. 6C–E). Overall, our results showed that MSCs and El induced K562 cells megakaryocytic differentiation through MPL and demonstrated the importance of JAK2/STAT3 and p38 MAPK signaling pathways in this process.

## Discussion

Chronic myeloid leukemia (CML) is characterized by the blocked differentiation process in the steps of hematopoiesis^30^. It is a myeloproliferative disorder in which an excessive accumulation of immature clonal myeloid cells in the blood and hematopoietic tissues is found^31^. For CML treatment, imatinib, a tyrosine kinase inhibitor (TKI), and its derivatives are considered as the most commonly used clinical approach^4^. Despite of improved outcomes in CML patients, less than 10% CML patients reach the stage of complete molecular response (CMR) and more than 60% of the patients who once achieved CMR eventually relapsed after TKI withdrawal^32^. A major obstacle to achieving durable CMR with TKI is the poor response in patients who progress to acceleration period (AP)/blastic crisis (BC) during which hematopoietic differentiation becomes blocked^33^. Hence, there is an urgent need to CML treatment by forcing malignant cells to undergo differentiation. In our study, we identified that inactivated MPL signaling arrested the CML cell megakaryocyte differentiation and contributed to the cancer progression. Allogeneic umbilical cord derived mesenchymal stem cells (UC-MSCs, hereafter called MSCs) restored the impaired MPL signaling and triggered megakaryocytic differentiation in CML cells. Furthermore, eltrombopag, a small molecule MPL agonist, exerted synergetic enhancement of effects on promoting CML cell differentiation, combined with MSCs. This combinatorial regimen significantly reduced the leukemia burden. The anti-leukemic activity of the combinatorial regimen may offer a promising therapeutic option for CML treatment.

Alterations of either TPO or MPL are involved in a variety of hematopoietic disorders^34,35^. The disability of MPL results in either thrombocytosis or thrombocytopenia^35^. This contradiction leads us to evaluate the function of the complex regulatory network between TPO/MPL axis and the downstream pathways. Previous studies have demonstrated that aberrant high expression of *MPL* was involved in the generation of AML^36–38^. However, the relationship between CML and TPO/MPL axis is still poorly understood. Our studies represented the first—to our knowledge—comprehensive expression pattern of *MPL* in CML cells. Compared to normal samples, the expression levels of *MPL* were significantly downregulated in CML patients. The *MPL* expression was inversely correlated to the progressive phases of the disease. Moreover, the gene set enrichment analysis (GSEA) revealed that the MPL signaling process, including JAK/STAT and MAPK pathways, were dramatically impeded. These data implied that the reversion of defects in MPL signaling might be beneficial to cure CML.

MPL is a homodimeric receptor of thrombopoietin, regulating proliferation in hematopoietic stem cells and megakaryocytic differentiation^39^. A study by Besancenot et al. showed that MPL and JAK2 expression levels controlled the balance between proliferation and differentiation in both physiological and pathological conditions^11^. When the MPL expression reaches a threshold, the MPL downstream signaling induced by TPO converts from proliferation to terminal maturation^11^. Therefore, it is assumed that increased *MPL* expression arrests malignant proliferation and promotes CML cell differentiation. In the present study, we demonstrated that the damaged expression of MPL in K652 cells could be restored by MSCs. Further, MSCs induced the megakaryocyte differentiation of CML cells by secreting TPO and subsequently activating the JAK/STAT and p38 MAPK signaling which has been acknowledged as differentiation-associated signaling. These beneficial effects of MPL suggested that MPL signaling may play an important role in regulating the proliferation/differentiation switch of leukemia cells.

We further postulated that the MSCs-induced activation of MPL and its downstream signaling may provide a fundamental for the combinatorial treatment with MPL agonist. Eltrombopag, a small molecule agonist of MPL^29^, was employed to extend the pro-differentiation effect of MSCs. Although eltrombopag treatment alone did not show any significant effect on megakaryocytic differentiation, MSCs in combination with eltrombopag exerted synergetic effects on promoting CML cell differentiation indicating that MSCs-mediated stimulation sensitized K562 cells to eltrombopag. Furthermore, the combinational treatment increased the MPL expression and stimulated the phosphorylation of JAK2, STAT3, and p38 MAPK in CML cells in vitro. Notably, the combination treatment in xenograft CML mouse model markedly ameliorated the CML burden and significantly eliminated CML cells in vivo. Thus, our data demonstrated a synergistic anti-leukemia efficacy of the drug combination, which provided an efficient pro-differentiation therapeutic strategy.

**Fig. 7:**
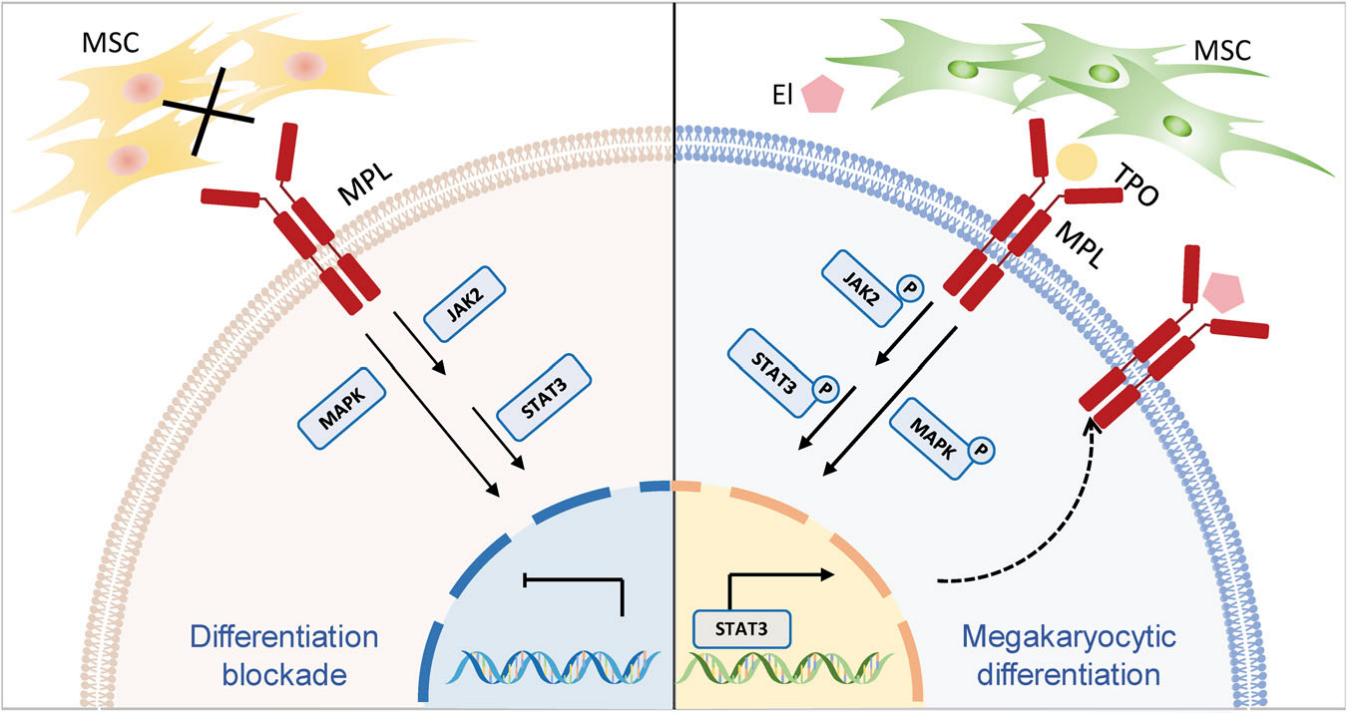
Schematic depiction of MSCs/El-mediated CML cells differentiation. CML cells with impaired expression of MPL cannot respond to differentiation signals (left), whereas allogeneic MSCs promoted leukemic cell differentiation via restoring damaged MPL signaling (right). MSCs secreted TPO to activate JAK2/STAT3 and p38 MAPK pathways, which may, in turn, increased MPL expression. El promoted higher phosphorylation of both JAK2/STAT3 and p38 MAPK. Arrows indicated activation; bar-headed lines indicated inhibition. Solid line denoted data from the present study or existing literature; dotted line denoted potential connections that require further documentation.

In conclusion, our report shed light on that impaired MPL signaling impeded the megakaryocytic differentiation in CML cells and contributed to the cancer progression. The overall schematic mechanism was illustrated in Fig. 7. Defective MSCs from CML patients maintained leukemia cells immature phenotype (left), whereas allogeneic MSCs restored normal MPL expression patterns, followed by the activation of the downstream signaling pathway of MPL (right). We showed that MSCs initiated the response of CML cells to MPL agonists. Hereby, MSCs in combination with eltrombopag triggered profound synergetic pro-differentiation effects through the phosphorylation of JAK2, STAT3, and p38 MAPK in CML cells. In concordance, the combination regimen significantly ameliorated the CML burden and synergistically executed differentiation-promoting effect in vivo. Consequently, our results provided a basis for expanding the clinical application of eltrombopag and paved the way to alternative CML therapeutic approaches aiming at the differentiation of CML cells.

## Supplementary information

supplemental figure 1

supplemental figure 2

supplemental figure 3

supplemental figure 4

supplemental figure 5

supplemental figure 6

supplemental figure 7

supplemental figure 8

supplemental table 1

supplemental figure legends
