# Supplementary material for: Establishment of a novel mesenchymal stem cell-based regimen for chronic myeloid leukemia differentiation therapy
Source: Cell Death Dis. 2021 Feb 24;12(2):208. doi: 10.1038/s41419-021-03499-w (PMC7904926; doi:10.1038/s41419-021-03499-w)
Supplement: Supplementary file 9 — supplemental table 1 [file 41419_2021_3499_MOESM9_ESM.docx]

| Gene name | Forward primer sequence  (5’-3’) | Reverse primer sequence  (5’-3’) |
| --- | --- | --- |
| GAPDH | GCACCGTCAAGGCTGAGAAC | TGGTGAAGACGCCAGTGGA |
| THPO | AACTGCAAGGCTAACGCTGT | GACATGGGAGTCACGAAGCA |
| MPL | CCCACTTTGGAACCCGATACG | GAGTCCGAGTCTGGTTTAGGA |
| CD41 | GATGAGACCCGAAATGTAGGC | GTCTTTTCTAGGACGTTCCAGTG |
| CD42b | CTGTGAGGTCTCCAAAGTGGC | GTGAGGCGAGTGTAAGGCATC |
| CD61 | GTGACCTGAAGGAGAATCTGC | CCGGAGTGCAATCCTCTGG |
| NFE2 | GCAGGAACAGGGTGATACAGC | GCAGCTCGGTGATGGACAT |
| RUNX1 | CTGCCCATCGCTTTCAAGGT | GCCGAGTAGTTTTCATCATTGCC |
| HBA1 | ACTGAACCTGACCGTACAACGCTGGCGAGT | AGCAGGCAGTGGCTTAGGAG |
| HBB | AGGAGAAGTCTGCCGTTACTG | CCGAGCACTTTCTTGCCATGA |
| HBE1 | ATGGTGCATTTTACTGCTGAGG | GGGAGACGACAGGTTTCCAAA |
| KLF1 | TTGCGGCAAGAGCTACACC | GTCAGAGCGCGAAAAAGCAC |
| Cyclin A | CGCTGGCGGTACTGAAGTC | GAGGAACGGTGACATGCTCAT |
| Cyclin D | GCTGCGAAGTGGAAACCATC | CCTCCTTCTGCACACATTTGAA |
| Cyclin E | AAGGAGCGGGACACCATGA | ACGGTCACGTTTGCCTTCC |
| CDK1 | AAACTACAGGTCAAGTGGTAGCC | TCCTGCATAAGCACATCCTGA |
| CDK4 | ATGGCTACCTCTCGATATGAGC | CATTGGGGACTCTCACACTCT |
| p21 | TGTCCGTCAGAACCCATGC | AAAGTCGAAGTTCCATCGCTC |
| p27 | AACGTGCGAGTGTCTAACGG | CCCTCTAGGGGTTTGTGATTCT |
| p53 | CAGCACATGACGGAGGTTGT | TCATCCAAATACTCCACACGC |
| MAP1LC3B | GATGTCCGACTTATTCGAGAGC | TTGAGCTGTAAGCGCCTTCTA |
| ATG4A | TGCTGGTTGGGGATGTATGC | GCGTTGGTATTCTTTGGGTTGT |
| ATG4B | ATGGACGCAGCTACTCTGAC | TTTTCTACCCAGTATCCAAACGG |
| ATG4C | TAGAGGATCACGTAATTGCAGGA | GTTGTCAAAGCTGAGCCTTCTAT |
| ATG4D | GGAACAACGTCAAGTACGGTT | CTCGCCCTCGAAACGGTAG |
| ATG5 | AAAGATGTGCTTCGAGATGTGT | CACTTTGTCAGTTACCAACGTCA |
| ATG7 | CAGTTTGCCCCTTTTAGTAGTGC | CCAGCCGATACTCGTTCAGC |
| FLI1 | CCAACGAGAGGAGAGTCATCG | TTCCGTGTTGTAGAGGGTGGT |
| ZFPM1 | CGTGCTTCGAGTGCGAGAT | GGCCTGAACAGTAGAGGCG |
| GATA1 | CTGTCCCCAATAGTGCTTATGG | GAATAGGCTGCTGAATTGAGGG |
| IRF7 | GCTGGACGTGACCATCATGTA | GGGCCGTATAGGAACGTGC |
| HIF1A | GAACGTCGAAAAGAAAAGTCTCG | CCTTATCAAGATGCGAACTCACA |
| ELF2 | AAACTGTAGTGGAGGTGTCAACT | CATGGCTATCTGGTGATGTTGG |
| SP3 | GCGACAGGTGATTTGGCTTCT | TACTGCCCACTTGAAGTAGCA |
| HNF4A | CACGGGCAAACACTACGGT | TTGACCTTCGAGTGCTGATCC |
| ETS2 | CCCCTGTGGCTAACAGTTACA | AGGTAGCTTTTAAGGCTTGACTC |
| NF1 | AGATGAAACGATGCTGGTCAAA | CCTGTAACCTGGTAGAAATGCGA |
