# Supplementary material for: Establishment of a novel mesenchymal stem cell-based regimen for chronic myeloid leukemia differentiation therapy
Source: Cell Death Dis. 2021 Feb 24;12(2):208. doi: 10.1038/s41419-021-03499-w (PMC7904926; doi:10.1038/s41419-021-03499-w)
Supplement: Supplementary file 10 — supplemental figure legends [file 41419_2021_3499_MOESM10_ESM.docx]

**Fig. S1** **CML progression was accompanied by decreased MPL expression.**

1. Expression levels of *MPL* in normal and CML Hematopoietic Stem Cells (HSCs), Multipotent Progenitor Cells (MPPs), Common Myeloid Progenitors (CMPs), Granulocyte-monocyte Progenitors (GMPs) and Megakaryocyte-erythroid Progenitors (MEPs).
2. *MPL* expression levels of CML cells in distinct phases (chronic phase, CP; accelerated phase, AP; blast crisis, BC) and different differentiation states (HSC, MPP, CMP, GMP and MEP).

Error bars indicated mean ± SEM (*P* values: two-tailed Student’ s *t*-test; * *P* < 0.05, ** < 0.01, *** < 0.001).

**Fig. S2** **Module containing MPL was negatively correlated to CML progression and differentiation arrest.**

1. Sample dendrogram and trait heatmap of the data involved in WGCNA. The colors represent the proportion to clinical diagnosis (Normal, CP, AP, BC).
2. Soft threshold selection process. Numbers in the plots indicated corresponding soft threshold powers. The approximate scale-free topology was attained at the soft-threshold power of 6.
3. Dendrogram of modules on the consensus correlation. Red line indicating the merging threshold, modules whose correlation greater than 0.7 were merged together.
4. Gene cluster dendrogram. Each color represented a module which contained a group of highly connected genes. A total of 25 modules were identified.
5. Relationships of consensus module and different phases of CML progression (Normal, CP, AP, BC). Each row in the table corresponded to a consensus module, and each column to a phase. The heatmap was colored by correlation according to the color legend (red, positively correlated; blue, negatively correlated).
6. GO enrichment analysis to the genes in yellow module. The result was visualized by upset plot.
7. Upstream transcriptional regulators identification of the genes in yellow module. The linkages between the top 10 enriched transcriptional regulators and their target genes were visualized in the network.

**Fig. S3 Treatment with MSCs resulted in** **megakaryocytic differentiation rather than erythroid differentiation.**

1. LAMA-84 cells were cultured alone or co-cultured with MSCs for different time. The relative mRNA levels of differentiation genes and transcription factors were assessed by RT-qPCR (n = 3).
2. Morphological changes accompanied by megakaryocytic differentiation were confirmed by Wright-Giemsa staining of cells with or without MSCs culture. Scale bars, 50 μm. Comparison of the cell size in LAMA-84 cells prior to or after stimulation by MSCs.
3. The amounts of cell surface protein CD41 and CD42b were measured by flow cytometric. The amounts of LAMA-84 cell surface proteins were expressed as the geometric mean fluorescence intensity.
4. K562 cells were cultured alone or co-cultured with MSCs for indicated time. The relative genes expression of erythroid differentiation genes was assessed by RT-qPCR (n = 3).
5. Flow cytometry analysis of the megakaryocytic differentiation surface markers CD41 and CD42b in K562 cells incubated with MSC for different time.

All statistical data in this figure showed the mean ± SEM (**P* < 0.05, ** < 0.01, *** < 0.001; one-way ANOVA was used for multiple comparisons).

**Fig. S4 MSCs inhibited the proliferation in K562 cells**

(A-B) K562 cells were co-cultured with MSCs for indicated time. Cell cycle (A), cell proliferation (B) were measured by flow cytometry.

(C) mRNA levels of the cell cycle and proliferation genes were measured by quantitative real-time PCR (n = 3).

All statistical data in this figure showed the mean ± SEM (**P* < 0.05, ** < 0.01, *** < 0.001; one-way ANOVA was used for multiple comparisons).

**Fig. S5 Induction of autophagy was involved in MSCs-mediated megakaryocytic differentiation.**

1. RT-qPCR of the expression of autophagy genes in K562 cells that were incubated with MSCs for the time indicated (n = 3).
2. K562 cell lysates from cells treated for various time with MSCs were analyzed by western blotting using anti-LC3B antibody. GAPDH was used as a loading control. Quantification of the protein level by using image J software (bottom).

(C) K562 cells were infected with 20 MOI Ad-GFP-LC3 for 24-72h, representative images of cells stained with GFP to label LC3. scale bar = 100 μm.

(D) K562 cells were treated in normal medium, glucose deprived or serum-free starvation condition for 48 h. The autophagy and differentiation genes were determined by RT-qPCR (n = 3).

(E) K562 cells were incubated for 48 h with MSCs in the presence or the absence of 3-MA (5 mM). The autophagy and differentiation genes expression were analyzed by RT-qPCR (n = 3).

(F) K562 cells were incubated for 48 h with MSCs in the presence or the absence of 3-MA (5 mM). The amounts of cell surface protein CD41 and CD42b were measured by flow cytometric.

(G) K562 cells were incubated for 5 days with MSCs in the presence or the absence of 3-MA (5 mM). Morphological changes were confirmed by Wright-Giemsa staining. Scale bars, 50 μm. The arrows indicated cytoplasmic vacuoles.

All statistical data in this figure represented the mean ± SEM (**P* < 0.05, ** < 0.01, *** < 0.001, ns, not significant, by one-way ANOVA in **A, B**and **D** and by Student’s *t* test in **E** and **F**). K: K562 cells; M: MSCs; 3-MA: 3-Methyladenine.

**Fig. S6 MSCs contributed to inducing megakaryocytic differentiation by activating MPL in CML cells.**

(A) The protein levels of MPL in membrane (MP) and cytosol (CP) were analyzed by western blotting. ATPase Na^+^ / K^+^ beta 2 (ATPase) was used as a loading control for membrane fraction, and GAPDH was used as a loading control for cytoplasmic fraction.

(B) Knockdown of MPL by transfection with MPL-siRNA was detected by Western blotting. β-Actin was used as a loading control.

(C) The si-NC- or si-MPL-transfected K562 cells were cultured alone or co-cultured with MSCs. After 48h, the differentiation markers in K562 cells were analyzed by RT-qPCR (n = 3).

(D) The phosphorylation status of ERK and AKT signaling pathways were determined by western blotting after treatment with MSCs for 48h. GAPDH was used as a loading control.

(E-F) The MPL overexpression efficiency of K562 or LAMA-84 cells (OE-K562 or OE-LAMA) was detected by western blotting and flow cytometric. GAPDH was used as a loading control.

(G) CD41 and CD42b levels of OE-LAMA cells were measured by flow cytometric after treatment with El for 48 h.

Results were represented as the mean ± SEM. *P* value was determined by Student’s *t* test (**P* < 0.05, ** < 0.01, *** < 0.001, ns, not significant). si-NC: siRNA negative control; si-MPL: MPL siRNA; OE-K562/OE-LAMA: MPL overexpressed K562 or LAMA-84 cells.

**Fig. S7 Combination treatment with MSCs and El triggered synergistically differentiation effects in LAMA-84 cells.**

1. The expression of surface markers CD41 and CD42b of K562 cells was measured by flow cytometric analysis and the mean fluorescence intensity was quantified.
2. Morphological changes in K562 cells that accompanied megakaryocytic differentiation were confirmed by Wright-Giemsa staining of cells in the presence and absence of El (0.5 μM, 1 μM and 2 μM) for 48 h. Scale bars, 50 μm.
3. K562 cells were treated with either MSCs or combination with El for 48 h, the expression levels of megakaryocytic differentiation transcription factors were assessed by RT-qPCR (n = 3).

All statistical data in this figure showed the mean ± SEM (**P* < 0.05, ** < 0.01, *** < 0.001; one-way ANOVA was used for multiple comparisons).

**Fig. S8 MSCs/El-mediated megakaryocytic differentiation was independent of activation of the AKT and ERK pathways.**

1. The protein levels of AKT and ERK signaling pathways and phosphorylation status were measured by Western blotting after treatment with either MSCs or El alone or the combination for 48 h. GAPDH was used as a control.

All statistical data in this figure showed the mean ± SEM (**P* < 0.05, ** < 0.01, *** < 0.001; one-way ANOVA was used for multiple comparisons).
